# Supplementary material for: Mechanism-based approach in designing patient-specific combination therapies for nonsense mutation diseases
Source: bioRxiv. 2024 Nov 14:2024.11.13.623453. Preprint. [Version 1] doi: 10.1101/2024.11.13.623453 (PMC11601491; doi:10.1101/2024.11.13.623453)
Supplement: Supplementary file 1 [file NIHPP2024.11.13.623453v1-supplement-1.pdf]

bioRxiv preprint doi: https://doi.org/10.1101/2024.11.13.623453; this version posted November 14, 2024. The copyright holder for this preprint (which was not certified by peer review) is the author/funder, who has granted bioRxiv a license to display the preprint in perpetuity. It is made available under aCC-BY 4.0 International license.

SUPPLEMENTARY DATA included below, consists of Figure S1 and Tables S1 – S2.

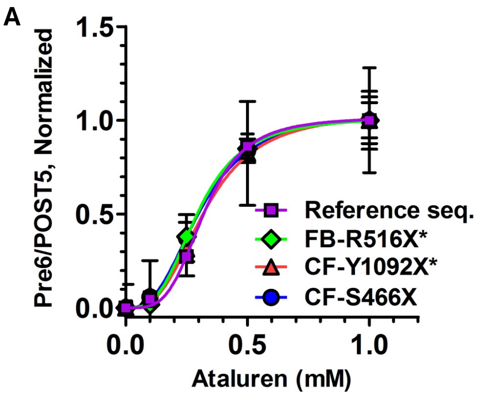

#	Stop-POST5	EC_{50}^{Ata}, mM	Hill n	PRE6/POST5
1	Reference	0.32 ± 0.02	3.6 ± 0.5	24 ± 5
2	CF-S466X	0.29 ± 0.07	2.8 ± 0.2	25 ± 6
3	CF-Y1092X^{A}*	0.33 ± 0.04	2.9 ± 0.3	33 ± 6
4	FB-R516X*	0.29 ± 0.03	3.1± 0.2	8.5 ± 2.3
Stop-POST5, 0.05 μM; RFC, 0.05 μM; Trp-TC 0.2 μM.				

Figure S1. ***A***. Similarity of dependence of readthrough on ataluren concentration for different mutant sequences. ***B.*** Parameter values.

**Table S1. Oligo-DNA sequences used in purifying isoacceptor tRNAs**		
tRNA	Codon/Anticodon	Biotin conjugated oligonucleotide sequence
Ec-Lys	UUU/AAA	5- AAA AGT CAA CTG CTC TAC CAA CTG AGC /3Bio/ -3'
Sc-Lys	UUU/AAA	5- TTA AAA GCC GAA CGC TCT ACC AAC TGA /3Bio/ -3
Ec-Val	GUG/TAC	5- GTA AGG GAG GTG CTC TCC CAG CTG AGC /3Bio/ -3'
Sc-Val	GUG/TAC	5- GTG TGA AGG CAA CGT GAT AGC CGC TAC /3Bio/ -3'
Sc-Arg	AGA/1CU	5- AGA AGT CAG ACG CGT TGC CAT TAC G /3Bio/ -3'
Ec-Gln	CAA/UUG	5- CAA AAA CCG GTG CCT TAC CGC TTG GCG /3Bio/ -3'
Sc-Gln	CAA/UUG	5- ATC AAA ACC GAA AGT GAT AAC CAC TAC /3Bio/ -3'
Sc-Trp	UGG/CCA	5- TTT GGA GTC GAA AGC TCT ACC ATT /3Bio/ -3'
Sc-Leu	CUA/UAG	5- CTA AAT CTG ACG CCT TAA ACC AC /3Bio/ -3'
Ec-Met	AUG/CAT	5- ATG AGT GAT GTG CTC TAA CCA ACT GAG C /3Bio/ -3'
Sc-Glu	GAA/UUC	5' - GTG AAA GCG TGA TGT GAT AGC CGT TAC /3Bio/ -3'
Sc-Leu	UUG/CAA	5' - GCT TGA ATC AGG CGC CTT AGA CCG CTC /3Bio/ -3'
Sc-Thr	ACU/AGU	5' - TTA CTA GTG TGG CGC CTT ACC AAC TTG /3Bio/ -3'
Sc-Lys	AAA/UUU	5' - TTA AAA GCC GAA CGC TCT ACC AAC TGA /3Bio/ -3'
Sc-Tyr	UAC/GUA	5' - ATT ACA GTC TTG CGC CTT AAA CCA ACT /3Bio/ -3'
Ec-Cys	UGC/GCA	5' - TGC AAT CCG CTA CAT AAC CGC TTT GTT AAC /3Bio/ -3'

bioRxiv preprint doi: https://doi.org/10.1101/2024.11.13.623453; this version posted November 14, 2024. The copyright holder for this preprint (which was not certified by peer review) is the author/funder, who has granted bioRxiv a license to display the preprint in perpetuity. It is made available under aCC-BY 4.0 International license.

Table S2. Rate constants for RFC interaction with Stop-POST5 complexes determined by smFRET experiments

Stop-POST5	Sequence downstream	$k_{arrival,app}$ (min^{-1})	$k_{heRF1\ dis}$ (min^{-1})	$k_{tRNA\ dis}$ (min^{-1})
Reference	UGA CUA AUG	1.63 ± 0.14	0.061 ± 0.005	0.065 ± 0.005
CF-S1196X	UGA CAC GUG	1.36 ± 0.19	0.049 ± 0.006	0.062 ± 0.006
CF-W1282X	UGA AGG AAA	1.50 ± 0.15	0.057 ± 0.007	0.065 ± 0.006
CF-R1162X	UGA CUC UUU	1.31 ± 0.18	0.039 ± 0.006	0.056 ± 0.007
FB-R516X	UGA GCU GGA	1.56 ± 0.13	0.043 ± 0.005	0.049 ± 0.005
FB-R2694X	UGA GGA AAC	1.23 ± 0.17	0.045 ± 0.008	0.059 ± 0.008
CF-R553X*	UGA GCA AGA	2.05 ± 0.13	0.014 ± 0.003	0.012 ± 0.006
CF-RQ30X	UAG CGC CUG	0.94 ± 0.13	0.034 ± 0.006	0.064 ± 0.007
CF-Y1092X[G]	UAG CUG UCA	1.41 ± 0.12	0.048 ± 0.006	0.055 ± 0.007
CF-Q685X	UAA UCU UUU	1.35 ± 0.13	0.051 ± 0.006	0.052 ± 0.006
CF-E92X	UAA GUC ACC	1.81 ± 0.14	0.038 ± 0.004	0.041 ± 0.006
